# No changes of cholesterol levels with a commercially available glucosamine product in patients treated with lipid lowering drugs: a controlled, randomised, open cross-over trial

**DOI:** 10.1186/2050-6511-13-10

**Published:** 2012-10-10

**Authors:** Robert Eggertsen, Åke Andreasson, Lennart Andrén

**Affiliations:** 1 Department of Medicine/Primary Health Care, University of Gothenburg, Mölnlycke vårdcentral, Ekdalavägen 2, S 435 30, Mölnlycke, Sweden; 2 Landvetter vårdcentral, Centrumhuset, S 438 32, Landvetter, Sweden; 3 Department of Clinical Pharmacology, University of Gothenburg, Sahlgrenska University Hospital, S 413 45, Göteborg, Sweden

## Abstract

**Background:**

The widespread use of natural health products is also a problem, as they could interact with prescribed drugs in patients. One commonly used product is glucosamine for osteoarthritis and some reports have found increased values of cholesterol and other lipids in patients treated with simvastatin for hypercholesterolemia. The aim of this trial was to investigate the effects of glucosamine on s-cholesterol levels (total s-cholesterol, s-HDL, s-LDL) in primary care patients on treatment with simvastatin or atorvastatin.

**Methods:**

Controlled, randomized, open, crossover pharmacodynamic study in two primary health care centres. Patients were treated with Artrox® (glucosamine) 625 mg twice daily and control (a commercially available multivitamin tablet Vitamineral®). The study started with a run-in period of four weeks followed by control or active treatment with randomization of sealed envelopes. Each treatment period was four weeks and the treatment with simvastatin or atorvastatin was unchanged during the study (12 weeks). 34 patients were treated with a stable dose of simvastatin (n=21) or atorvastatin (n=13) for at least three months. Assessments of total s-cholesterol, s-HDL, S-LDL and s-triglycerides were performed in the morning with the patients in a fasting condition. T-tests for paired samples were used for statistical analyses and a p-value <0.05 was considered significant. Endpoints were the differences in lipid values at week 8 and week 12.

**Results:**

All patients completed the study. No significant changes were seen on any of lipid levels in the simvastatin group**.**

**Conclusion:**

The actual glucosamine product did not change lipid levels of patients treated with simvastatin. Atorvastatin group was too small for safe calculations but was also without changes**.**

**Trial registration:**

EUDRACT2006-001458-28

## Background

There is a widespread use of natural health products [1,2]. It is also known that some of these products can interact with several prescribed drugs [3], causing increased levels for example of cholesterol and other lipids [4,5] when interacting with the metabolism of simvastatin and atorvastatin [4,5]. One drug that is commonly used of patients suffering from osteoarthritis is glucosamine, which is found naturally in the body and also is used by the body as one of the building blocks of cartilage. Some case reports have found increased values of cholesterol and other lipids in patients treated with simvastatin and glucosamine [6] but no prospective studies have been conducted to confirm these data. One long-term study of glucosamine effects on osteoarthritis found no effects on lipids but this was not made on patients treated with simvastatin [7]. One Danish trial found similar results but these patients were not either treated for hypercholesterolemia [8]. We have found effects on patients treated with simvastatin and atorvastatin of St John´s Wort as a consequence of interaction of the CYP3A4 (4,5). In one trial on healthy subjects, glucosamine was tested together with noskapin where no such effects could be demonstrated for glucosamine [9]. Artrox® is a widely used commercially available product containing 750 mg glucosaminehydrochlorid that is equivalent with 625 mg glucosamine. The recommended daily dose is 625 mg given twice daily. Patients in primary health care often ask their doctor of possibilities to take natural remedies or over-the counter drugs together with prescribed medicines. It is therefore important to investigate possible interactions to prevent negative effects and give right advice to the patients. Simvastatin and atorvastatin are to a large extent metabolized by cytocrom P450 3A4. The hypothesis for our study was that glucosamine could interfere with this major metabolic pathway causing a deterioration in lipid control as was reported for three cases in Denmark (8). Against the background that no trial is carried out on patients on treatment for high levels of lipids, we decided to investigate the influence of the commercially available glucosamine product Artrox® on relevant pharmacodynamic interactions in patients with hypercholesterolemia, treated with simvastatin or atorvastatin in a randomized, controlled crossover study.

## Methods

There were totally 21 patients with hypercholesterolemia on treatment with a stable dose of simvastatin (10–40 mg once daily) and 13 patients with atorvastatin (10–20 mg daily) that were included in the study. The 11 males and 10 females in the simvastatin group had a mean age of 66 years, range 57–77 years. Fourteen patients had hypertension, nine type 2 diabetes and five had suffered from cardiac infarction or stroke (Table 1). In the atorvastatin group there were nine men and four women with a mean age of 67 years, range 47–75 years. Eight patients had hypertension, five had diabetes, and four had earlier experienced heart infarction or stroke (Table 1).

**Table 1:** patients characteristics

**Simvastatin treatment n = 21**		
		Mean
Dose mg	10-40	21
Age years	57-77	66
Male n	11	
Female n	10	
Weight kg	47-110	83
Length cm	157-185	169
Hypertension n	14	
Diabetes n	9	
Heart disease/stroke n	5	
Systolic blood pressure mm Hg	110-178	169
Diastolic blood pressure mm Hg	60-90	78
Heart rate b/min	46-80	62
Atorvastatin treatment n = 13		
		Mean
Dose mg	10-20	15
Age years	47-75	67
Male n	9	
Female n	4	
Weight kg	60-105	87
Length cm	162-181	170
Hypertension n	8	
Diabetes n	5	
Heart disease/stroke n	4	
Systolic blood pressure mm Hg	109-165	142
Diastolic blood pressure mm Hg	64-100	82
Heart rate b/min	50-76	63

The study was approved by the Swedish Medical Products Agency and the Regional Ethical Board in Western Sweden.

The study was performed as a randomised, open, cross-over study, where the approved glucosamine containing product Artrox® was compared with an inactive control (a commercially available vitamin product named Vitamineral®). Patients were recruited from the primary health care centres in Mölnlycke and Landvetter in the Western region of Sweden. Eligible patients on simvastatin or atorvastatin treatment were identified by the routinely used data system at the primary health care centres. Patient characteristics are given in Table 1. Patients were invited by an introductory letter which included information about the study. The information leaflet was approved by the regional ethical board and it was sent home to eligible patients. Those who were willing to participate signed an informed consent and were included in the study. No record was made of those patients who did not respond to the information letter.

Exclusion criteria were; unstable angina pectoris, recent myocardial infarction (within a year), recent stroke (within a year), cardiac failure, HIV and dementia. There have not been shown any significant interactions involving glucosamine except case reports to warfarin treated patients. However, we excluded same drugs as in our earlier trials with St John´s Wort as our hypothesis was a stimulation of CYP3A4. Thus, treatment with birth control pills, warfarin, theofylline, cyclosporine, amitryptilin, nortriptilin, digoxin or sertraline was not allowed.

The study started with a four week run-in period. At the initial visit (week 0), the patients were informed about the study, inclusion and exclusion criteria were verified and a physical examination was performed. All blood samples were obtained in the fasting condition and analysed at the accredited central laboratory at Sahlgrenska University Hospital in Gothenburg (total s-cholesterol, s-HDL-cholesterol, s-LDL-cholesterol and s-triglycerides). The laboratory was unaware of patient treatment. Patients were then to return after 4 weeks (week 4) and another physical examination was performed and blood samples as described at week 0 above were collected. At this visit, the treating physician randomised (using sealed envelopes performed by a person not involved with patients), the order of treatment with active (Artrox®) and control (Vitamineral®). Thus, 50% started with active treatment and the other 50% started with control treatment and all were crossed- over to the other treatment modality after another 4 weeks (week 8). We considered that the treatment period o four weeks was adequate for possible glucosamine effects to vanish and thus no further wash out period was mandatory. Assessment of total s-cholesterol, s-HDL and s-LDL-cholesterol and s-triglycerides were then performed at the end of each treatment period (week 8 and 12). Compliance was verified by pill counting at each visit. The patients were on their regular dose of simvastatin or atorvastatin and the dose of simvastatin and atorvastatin was kept unchanged during the whole study period until all patients completed the study. The mean dose of simvastatin was 21 mg (range 10 mg – 40 mg) and of atorvastatin 14.6 mg (range 10–20 mg).

Glucosamine could have an effect on glucose metabolism as stated in the SPC of the product. In order to exclude negative effects on this variable, HbA1c was followed as a safety measure,

### Statistics

T- test for paired samples was used for statistical analyses and a p-value <0.05 was considered significant. The primary efficacy endpoint was the difference in LDL-cholesterol at week 8 and 12 between active treatment and control. Secondary endpoints were the corresponding difference in total cholesterol, HDL- cholesterol and in triglycerides**.** The study was powered to detect a difference of 0.48 mmol/L with a power of 81.2 % with a significance level of 0.05 (two sided) and a standard deviation within the groups of 0.52. Power calculations showed that participation of 20 subjects would give statistical significance. Randomization procedure was performed by a person not involved with the patients.

## Results

### Simvastatin group

Basal measurements were made after four weeks of run-in. There were no significant effects on s-LDL, s-HDL, total s-cholesterol, or s-triglycerides after four weeks treatment with glucosamine. Neither were there any significant changes of p-glucose and HbA1c Table 2.

**Table 2:** Effects on lipid and glucose values

	**Vitamineral mean and SD**	**Simvastatin mean and SD**	**P-value**
s-LDL mmol/L	2.27+0.61	2.30+0.51	0.81 ns
s-HDL mmol/L	1.62+0.55	1.63+0.60	0.86 ns
s-total cholesterol mmol/L	4.69+0.99	4.76+0.96	0.53 ns
s-triglyceridesmmol/L	1.77+0.76	1.83+0.79	0.63 ns
p-glucose mmol/L	6.77+1.90	6.90+1.62	0.70 ns
HbA1c mmol/mol	5.13+1.10	5.09+0.97	0.57 ns
		Atorvastatin	
s-LDL mmol/L	2.36+0.65	2.36+0.64	1.00 ns
s-HDL mmol/L	1.55+0.40	1.48+0.40	0.10 ns
s-total cholesterol mmol/L	4.51+0.65	4.40+0.89	0.42 ns
s-triglyceridesmmol/L	1.74+1.05	1.59+0.70	0.39 ns
p-glucose mmol/L	7.39+2.08	7.53+2.16	0.38 ns
HbA1c mmol/mol	5.20+1.13	5.18+1.40	0.90 ns

### Atorvastatin group

This group (n=13) was too small for safe calculations but also in this group there were no effects on the different lipids that could be demonstrated when compared with run-in determinations and after four weeks of active treatment. Values of p-glucose and HbA1c were also unchanged Table 2.

All patients completed the study and no significant side effects were reported.

## Discussion

The main finding in our study was that we could not demonstrate any significant effect on total s-cholesterol, s-LDL, S-HDL or s-triglycerides after treatment with the glucosamine product Artrox® on patients treated with neither simvastatin nor atorvastatin for increased levels of lipids. However, the atorvastatin group comprised of 13 patients why safe conclusions could not be drawn from these patients. This is valuable knowledge as the use of glucosamine is widespread and a considerable amount of people is now also treated with lipid lowering drugs.

Many patients are not aware of possibilities for interaction with increasing lipid values and different natural health products that you can buy on the counter [4,5]. We have used one commercial product in our trial but results may be similar with other glucosamine products Also positive effects are described of natural health products as for pomegranate preparations where reductions in LDL-cholesterol have been demonstrated [10,11]. Such products are as a rule not tested for influence on different metabolic interactions, why it is important to make investigations in that field. There are made meta-analysis of the effects of glucosamine but reports of interactions and effects of lipids are missing [12,13]. Reports of glucose rising and induction of insulin resistance of glucosamine have been discussed but not been confirmed in clinical studies [14]. The Swedish Medical Products Agency (MPA) points out that interaction studies are lacking and precaution is important even if the risks of interactions is estimated as low [14]. In our material we could not find any disturbances in the glucose metabolism, however the active treatment period was only four weeks.

A limitation in our study is that it was not double-blind, why we cannot exclude carry-over effects in the cross-over regimen. However, the half life for glucosamine is about two hours why this seems unlikely in treatment periods of four weeks. Furthermore, the study was randomized, and assessment of the lipids was performed by an accredited central laboratory, blinded to the treatment regimen. We can not with used sample sizes definitely exclude that there may be a type 2 error because our study was dimensioned to detect differences between the groups. Further studies with larger materials could be necessary. The metabolism of glucosamine is not known. This was an explanatory study to investigate if there was a signal that could verify clinical observations of possible interactions. If a signal had been detected further studies on possible mechanism had been justified.

## Conclusions

We conclude that no increased levels of lipids were seen in patients on lipid lowering drugs as simvastatin if they simultaneously take glucosamine for articulation problems.

## Competing interests

None of the authors have any relevant conflicts of interest.

## Authors’ contributions

All authors planned and managed the study, performed the analysis and wrote the main results and final manuscript. All authors read and approved the final manuscript.

## Pre-publication history

The pre-publication history for this paper can be accessed here:

http://www.biomedcentral.com/2050-6511/13/10/prepub
